# Supplementary material for: Defining the 3′Epigenetic Boundary of the FMR1 Promoter and Its Loss in Individuals with Fragile X Syndrome
Source: Int J Mol Sci. 2023 Jun 27;24(13):10712. doi: 10.3390/ijms241310712 (PMC10342127; doi:10.3390/ijms241310712)
Supplement: Supplementary file 1 [file ijms-24-10712-s001.zip › ijms-2402687-supplementary.pdf]

Defining a novel 3′epigenetic boundary of the *FMR1* promoter lost in individuals with fragile X syndrome.

Supplementary Online Content

Note S1. Genomic organization and 5' *FMR1* targeted regions.

Figure S1. Xq27.3 sequence organization and specific CpG site locations on GenBank L29074 in relation to regions targeted by EpiTYPER system based methylation analysis.

Figure S2. The CG dinucleotide density in the regions proximal to the FREE2 3' Boundary.

Figure S3. Intergroup comparison heat-maps of P values for the raw data presented in Figure 2 of CpG unit specific methylation in male controls CGG<40 (HC n=20), males with premutation (PM n=39), FM with developmental delay and 100% methylated by Southern blot (FM n=73), FM CGG size mosaics (PM/FM n=27), FM methylation mosaics (FM Met. Mos. n=21) and 'high functioning' males (IQ>70) with an unmethylated FM (UFM n=5).

Figure S4. Intergroup comparison heat-maps of P values for the raw data presented in Figure 3 of CpG unit specific methylation in female controls CGG<40 (n=75), females with a PM (n=157), FM (n=132), as well as females mosaic for PM and FM alleles (n=8).

Table S1. Nine CpG units overlapping between amplicons FREE2 (B), (C) and (D).

Table S2. Putative regulatory motifs proximal to the *ASFMR1* transcription start site within the FREE3 region.

Table S3. Mann-Whitney test P-value comparison matrix between males with *FMR1* expansions and control groups plotted in Figure 4.

Table S4. Sample size, median and interquartile range (IQR) for selected FREE2 and FREE3 CpG units from Figure 4 and Table S3.

Table S5. Amplicon details used for methylation analysis using the EpiTYPER system of the regions proximal to the CGG expansion at the Xq27.3 locus.

Table S6. Details of the post-mortem brain tissue processing, age and the clinical phenotype.

Note S1

Genomic organization and 5’ *FMR1* targeted regions.

For cross validation purposes the assays were designed so that there was overlap between CpG units on the 5’ and 3’ ends on most amplicons (Table S1). Based on this, the EpiTYPER system-based analysis in venous blood DNA provided methylation ratio values for 2 CpG units within *FMR1* exon 1 and 43 CpG units within *FMR1* intron 1 and 2. Of these 7 units overlapped between amplicons FREE2(B), FREE2(C) and FREE2(E), and consistently provided identical results for the overlapping units. Considering this study targeted a total of 34 novel CpG units within the 5’ portion of the *FMR1* intron 1, a total of 22 units were defined as novel epigenetic biomarkers differentially methylated in blood DNA within intron 1 between individuals with *FMR1* expansions and control group in males and females. These units encompassed 35 CpG sites that have not been previously examined and defined as fragile X related biomarkers in venous blood DNA. We found that the FREE2 region spanned ~1.3 kilobases downstream from the exon1/intron1 boundary to a novel 3’ epigenetic boundary identified within the FREE2(E) amplicon. The position of this boundary in venous blood DNA was identical to that in lymphoblast cell lines described in our previous work [16].

In the *FMR1* intron 2 we have examined methylation of the 2 CpG sites denoted as FREE3 (Figure 1). These sites flank *ASFMR1* transcription start site on either side with a TATA box immediately upstream of the FREE3 CpG1 [Figure S1 and Figure S2]. In our previous work both FREE3 (F3)CpG1 and (F3)CpG2 were hypomethylated in FXS cell lines that had significantly reduced *ASFMR1* expression compared to those from controls and individuals with unmethylated full mutation (UFM) alleles [16], suggesting that decreased methylation of these sites is involved in down regulation of *ASFMR1* expression in FXS. This gene regulation hypothesis is consistent with previous reports of methylation changes in close proximity to a TATA box being involved in regulation of gene expression originating from nearby transcription sites [38].

(13758) - **CGG expansion** – (13862)

FREE2(A) 5' Intr 1

(13951) fP(200-400)

ctgaagagaa gatggaggag ctggtggtgg aagtg**c1g**ggg ctccaatgg**c2g**ctttctacaag >> gtacttgg ctctagggca ggccccatct t**c3g**cccttcc ttccctccct tttcttcttg
gtgt**c4g**g**c5g**ggaggcaggcc **c6g**gggccctc ttcc**c7g**agca c**c8g**c**9g**cctgg gtgccagggc ac10gctc11ggc12g ggatgttgtt gggagggaag gactggactt gggcctgtt
ggaagcccct ct

(14192)

FREE2(B) 5' Intr 1

(14,167) rP(200-400) fP(400-600)

acttggggcc tgttggaagc ccctctc**c1g** actc**c2g**aga ggccctag**c3** gcctat**c4g**a aatgagagac c << >> ag**c5g**agga gagggttctc ttt**c6g**g**c7g** c**c8g**agccc**c9g**
c**c10g**gggtg agctggggat ggg**c11g**agg gc**c12g**g**c13g** gcaggtacta gagc**c14g**gg **c15g**ggaagg gc**c16g**aaat **c17g**g**c18g**c taagtga**c19g** g**c20g**atggc

fP(600-800) rP(400-600)

ttattccccc tttcctaaac atcatctccc ag**c21g**ggat c**c22g**ggcc>>t gt**c23g**tgtg ggtagttgtg g<<aggag**c24g** ggggg**c25g**c ttcagc**c26g** ggc**c27g**cct cctgcag**c28g**
ccaagagggc ttcaggtctc ctttggcttc tcttttc**c29g** gtctagcatt gggactt**c30g** gagagctcca ctgttctggg **c31g**agggctgt gaagaaagag tagtaagaag

fP(800-1000) rP(600-800)

C32ggtagt**c33**>>gg caccaaatca caatggcaac tgatttttag tggct<<tct

(14,620)

FREE2(C) 5' Intr 1

(14,466)

caaga gggcttcagg tctcctttgg cttctctttt c**c1g**gtctagc attgggactt **c2g**gagagctc cactgttctg gg**c3g**agggct gtgaagaaag agtagtaaga ag**c4g**gtagt**c5g** gcaccaaat
cacaatggca actgattttt agtggcttct ctttgtggat tt**c6g**gaggag attttagatc caaaagtttc aggaagacc ctaacatggcc cagcagtgca ttgaagaagt tgatcat**c7g**t
gaatatt**c8g**c**9**tcccccttt ttgttaaa**c10g** gggtaaattc aggaatgcac atgcttc

(14,767)

FREE2(D) 5' Intr 1

(14,652)

aaaagtttc aggaagacct aacatggcc cagcagtgca ttgaagaagt tgatcat**c1g**t gaatatt**c2g**c**3G**tccccccttt ttgttaaa**c4g** gggtaaattc aggaatgcac atgcttcag**c5g**

fP(1000-1200) rP(800-1000)

Tctaaaa>>ccattagcag**c6g** ctgct<<actta aaaattgtgt gtgtgtgttt aagtttccaa agacctaaat atatgccatg aaacttcagg taattaactg agagtatatt attactaggg

rP(1000-1200)

cattttttt ttaactgag**c7g** aaaatattt ttgtgcccct aagaacttg accacatttcc tttgaatttg tggtgttgca gtggactgaa ttgttgaggc t<<ttatatagg cattcatggg tttactgtgc
ttttt

(14,880)

2 CpG sites not analysed

(15,025) aaagt tacaccattg cagatcaact aacacctttc agttttaaaa ggaagattta caaatttgat gtagcagtag tg**cg**tttgtt ggtatgtagg tgctgtataa attcatctat aaattctcat
ttccttttga atgtctataa cctctttcaa taatatccca ccttactaca gtattttggc aatagaaggt g**cg**tgtggaa ggaaggctgg aaaatagcta ttagcagtgt ccaacacaat tcttaaatgt
attgta (15,646)

(15,280)

FREE2(E) 5' Intr 1

gaat ggcttgaatg tttcagacag gaca**c1g**tttg gctataggaa aataaacaat tgactttatt ctgtgtttac caattttatg aagacatttg gagatcagta tatttcataa atgagtaaag
tatgtaaact gttccatact ttgagcacaa agataaagcc ttttgctgta aaaggaggca aaaggtaacc c**c2g**c**3g**tttat gttcttaaca gtctcatgaa tatgaaattg tttcagttga ctctgcagtc
aaaattttaa tttcattgat tttattgatc cataatttct tctggtgagt ttg**c4g**tagaa tcgttcac**5g**g tcctagatta gtggttttgg tcactagatt tctggc

(15,646)

FREE3

(23,701)

tttttcttac acaggcattt gtaaaggatg ttcatgaaga ttcaataaca gttgcatttg aaaacaagta agtgtct**c1g**t taTATAATTt taatgatgag gttctttaat atTTTATGCT AATTctattc
ttcatttttt aaaaattcaa gtccagtttg agtgcttttc aggaatggat cttcatgtta ctgactgaga agtttctgaa caactcagta ttaaactaat ** ggaatgactg tttctgctaa tgtcctggag
gtcccttatt gtatggtatt gatcctt**ac2g** tcttaattcc cttgaatgtg aagaaagaaa ccagaga

(24,027)

Figure S1. Xq27.3 sequence organization and specific CpG site locations on GenBank L29074 L38501 in relation to regions targeted by EpiTYPER system-based methylation analysis. Primers utilized for EpiTYPER system based methylation analysis targeted 6 regions at the Xq27.3 locus designated as FREE2(A); FREE2(B); FREE2(C); FREE2(D); FREE2(E), FREE2(F) and FREE3 (color coded as in Figure 1). Individual CpG sites within each region are numbered accordingly. Motifs proximal to the *ASFMR1* transcription start site within the FREE3 region sites are indicated in capital font and are listed/identified in Table S2. ****** in red indicates *ASFMR1* transcription start site. The

FREE2 3’ boundary located between CpG1 and CpG2/3 of FREE2(E) which is underlined in the sequence and highlighted in red. Text highlighted in pink indicates CTCF binding sites from UCSF Chip-Seq: CTCF site #1 binds FREE2 (A)CpG10-12 to FREE2 (B) CpG32; CTCF site #2 binds FREE2(D) CpG5-7. >> and << symbols indicate forward and reverse primers used in ChiRP to show portion of FMR1 intron 1 that binds RNA in con and FXS human embryonic stem cells (hESCs) at day 45 of differentiation (Figure 4 and Figure S16 and Table S1 in [21]).

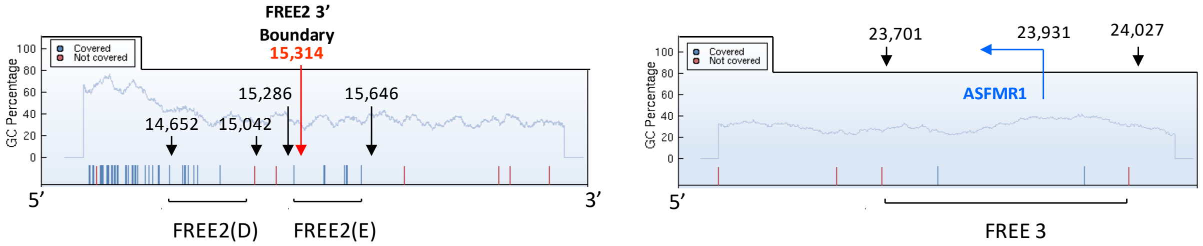

Figure S2. The CG dinucleotide density in the regions proximal to the FREE2 3' Boundary. The CpG sites that have been analysed for methylation status are represented in blue on the X axis, while sites not covered by our assays are represented in red. The coordinates of the FREE2(D) and FREE2(E) assays on the GenBank L29074 are also indicated.

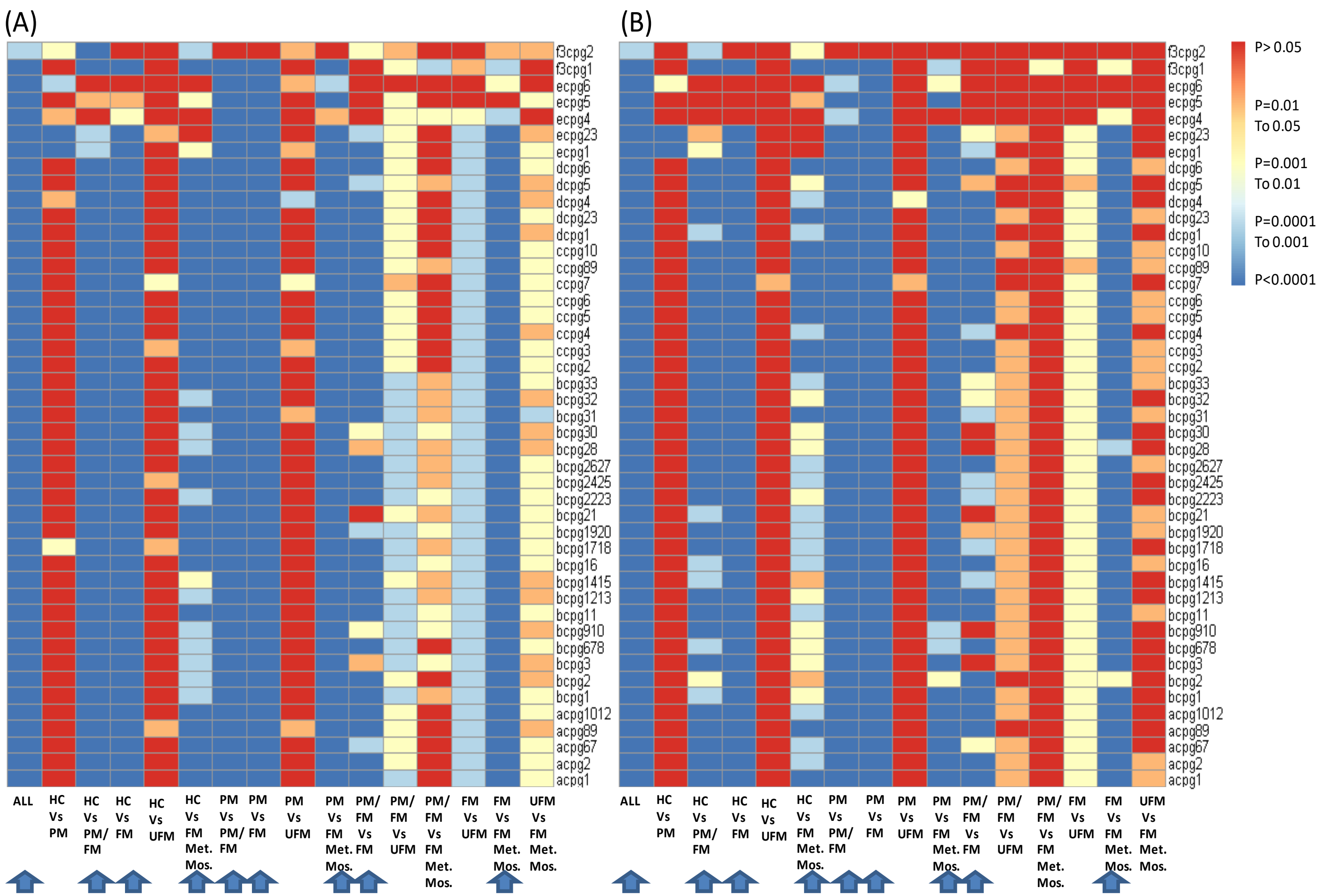

Figure S3. Intergroup comparison heat-maps of P values for the raw data presented in Figure 2 of CpG unit specific methylation in male controls CGG<40 (HC n=20), males with premutation (PM n=39), FM with developmental delay and 100% methylated by Southern blot (FM n=73), FM CGG size mosaics (PM/FM n=27), FM methylation mosaics (FM Met. Mos. n=21) and 'high functioning' males (IQ>70) with an unmethylated FM (UFM n=5). Each row indicates a different CpG unit, with bottom rows representing the 5' and top rows representing the 3' CpG units. Each column represents a different intergroup comparison. The P value heat-maps for **(A)** represent unadjusted comparisons, and **(B)** represents adjusted comparisons using bonferroni correction method for multiple testing, where the number of tests was equivalent to the number of pairwise comparisons. Note: The blue arrows indicate the most consistently significant P values for inter-group comparisons between CpG units and between comparisons "unadjusted" and "adjusted" for multiple comparisons.

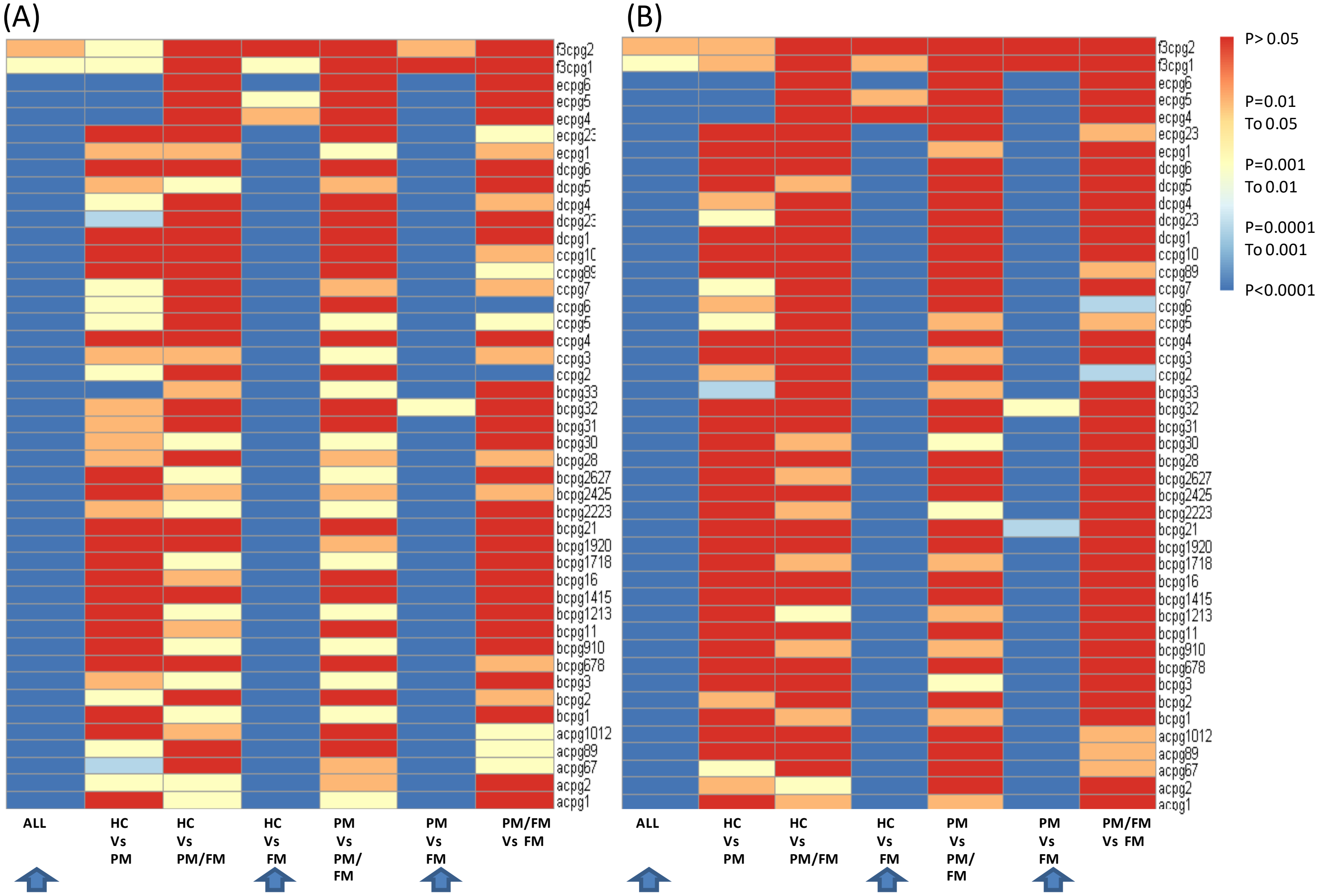

Figure S4. Intergroup comparison heat-maps of P values for the raw data presented in Figure 3 of CpG unit specific methylation in female controls CGG<40 (n=75), females with a PM (n=157), FM (n=132), as well as females mosaic for PM and FM alleles (n=8). Each row indicates a different CpG unit, with bottom rows representing the 5’ and top rows representing the 3’ CpG units. Each column represents a different intergroup comparison. The P value heat-maps for **(**A) represents unadjusted comparisons, and (B) represents adjusted comparisons using bonferroni correction method for multiple testing, where the number of tests was equivalent to the number of pairwises comparison. Note: The blue arrows indicate the most consistently significant P values from inter-group comparisons between CpG units and between comparisons “unadjusted” and “adjusted” for multiple comparisons.

Table S1. Nine CpG units overlapping between amplicons FREE2(B), (C) and (D).
*Note: Each row represents identifications of the overlapping CpG sites.

5'Amplicon ID:	CpG unit ID:	3'Amplicon ID:	CpG unit ID:
FREE2B	29 (not examined)	FREE2C	1 (not examined)
FREE2B	30	FREE2C	2
FREE2B	31	FREE2C	3
FREE2B	32	FREE2C	4
FREE2B	33	FREE2C	5
FREE2C	7	FREE2D	1
FREE2C	8.9	FREE2D	2.3
FREE2C	10	FREE2D	4

Table S2. Putative regulatory motifs proximal to the *ASFMR1* transcription start site within the FREE3 region.

**Transcription factor sites/potential regulatory motifs**	**Sequence on the sense strand**	**Strand**	**Sequence homology (%)**	**Amplicon #**	**CpG unit location**
SRY	AAACAAA	LEADING	100	5' of FREE3	5' of FREE3 CpG1
CdxA	TATAATT	LEADING	99	FREE3	CpG1
Oct-1	TTTATGCTAATT	LEADING	99	FREE3	Between CpG1 and CpG2

Table S3. Mann-Whitney test P-value comparison matrix between males with *FMR1* expansions and control groups plotted in Figures 4 and 5.

		(E)CpG1					
		HC	PM	UFM	FM only	FM sm	FM meth
	HC		< 0.0001	0.2751	< 0.0001	0.0003	0.0041
	PM	< 0.0001		0.0384	< 0.0001	< 0.0001	< 0.0001
(E)							
CpG2.3	UFM	0.0104	0.1881		0.0002	0.0050	0.0097
	FM only	< 0.0001	< 0.0001	0.0002		< 0.0001	< 0.0001
	FM sm	0.0010	< 0.0001	0.0032	0.0002		0.1042
	FM meth	0.1589	< 0.0001	0.0134	< 0.0001	0.0739	

		(E)CpG4					
		HC	PM	UFM	FM only	FM sm	FM meth
	HC		< 0.0001	0.2751	< 0.0001	0.0003	0.0041
	PM	< 0.0001		0.0384	< 0.0001	< 0.0001	< 0.0001
(A)							
CpG10-12	UFM	0.0104	0.1881		0.0002	0.0050	0.0097
	FM only	< 0.0001	< 0.0001	0.0002		< 0.0001	< 0.0001
	FM sm	0.0010	< 0.0001	0.0032	0.0002		0.1042
	FM meth	0.1589	< 0.0001	0.0134	< 0.0001	0.0739	

		(F3)CpG1					
		HC	PM	UFM	FM only	FM sm	FM meth
	HC		0.0646	0.1162	< 0.0001	< 0.0001	< 0.0001
	PM	0.0049		0.3168	< 0.0001	< 0.0001	< 0.0001
(F3)							
CpG2	UFM	0.2449	0.0400		0.0150	0.0096	0.2400
	FM only	0.2528	0.0581	0.1813		0.1440	0.0002
	FM sm	< 0.0001	0.2055	0.0206	0.0036		0.0005
	FM meth	0.0003	0.8100	0.0300	0.0445	0.2929	

Note: HC= male controls; PM=permutation; UFM = unmethylated FM by southern blot; FM only = only FM alleles detected 100% methylated by Southern blot; FM sm = FM CGG size mosaics; FM meth = FM methylation mosaics. Upper triangle in red represent the intergroup comparison p-values for (E)CpG1, (E)CpG4, (F3)CpG1. Lower triangle in blue represent the intergroup comparison p-values for (E)CpG2.3, (A)CpG10-12, (F3)CpG2.

Table S4. Sample size, median and interquartile range (IQR) for selected FREE2 and FREE3 CpG units from Figures 4 and 5 and Table S3.

	N	Median	IQR	n	Median	IQR
		(E)CpG1			(E)CpG2.3	
All	163	0.650	0.625	178	0.918	0.285
HC	15	0.220	0.100	19	0.740	0.075
PM	32	0.103	0.033	36	0.590	0.093
UFM	5	0.170	0.075	5	0.655	0.060
FM only	69	0.790	0.115	73	0.950	0.020
FM CGG size mosaics	22	0.658	0.345	24	0.930	0.090
FM methylation mosaics	20	0.423	0.425	21	0.820	0.220
		(E)CpG4			(A)CpG10-12	
All	177	0.970	0.030	171	0.500	0.760
HC	18	0.970	0.030	17	0.015	0.020
PM	38	0.950	0.035	39	0.020	0.014
UFM	5	0.955	0.020	5	0.020	0.005
FM only	72	0.980	0.020	66	0.798	0.105
FM CGG size mosaics	23	0.970	0.025	24	0.615	0.408
FM methylation mosaics	21	0.965	0.020	20	0.258	0.560
		(F3)CpG1			(F3)CpG2	
All	180	0.888	0.110	177	0.865	0.045
HC	20	0.968	0.023	18	0.880	0.025
PM	39	0.958	0.033	39	0.858	0.063
UFM	5	0.940	0.040	5	0.900	0.025
FM only	72	0.850	0.053	69	0.877	0.050
FM CGG size mosaics	25	0.835	0.083	27	0.855	0.060
FM methylation mosaics	19	0.910	0.065	19	0.855	0.030

Note: HC= healthy controls; PM=permutation; UFM = unmethylated FM by southern blot; FM only = pure FM 100% methylated by Southern blot.

Table S5. Amplicon details used for methylation analysis using the EpiTYPER system of the regions proximal to the CGG expansion at the Xq27.3 locus.

**Amplicon ID:**	**PCR conditions:**	**Size (bp)**	**Distance (bp) from:**	**Primer sequence:**
**FREE2 (A)**	15 mins at 95°C; 10 cycles of: 94ºC for 30s, 56ºC for 30s, 72ºC for 1min; 35 cycles of: 94ºC for 30s, 62ºC for 30s, 72ºC for 1min; 72ºC for 7min	242	89 – 3'(CGG)n	Fw: 5'- AGGAAGAGAGTTGAAGAGAAGAT GGAGGAGTTGG -3' Rv:5'-CAGTAATACGACTCACTATAGGGAGAAGGCT AAAAAAACTTCCAACAAACCCC -3'
**FREE2 (B)**	15 mins at 95°C; 10 cycles of: 94ºC for 20s, 54.1ºC for 30s, 72ºC for 1min; 35 cycles of: 94ºC for 20s, 60.1ºC for 30s, 72ºC for 1min; 72ºC for 7min	454	330 – 3'(CGG)n	Fw : 5'-AGGAAGAGAGATT TGG GGT TTG TTG GAA GTT T-3' Rv: 5'- CAGTAATACGACTCACTATAGGGAGAAGGCTAAA AAC CAC TAA AAA TCA ATT ACC A-3'
**FREE2 (C)**	15 mins at 95°C; 10 cycles of: 94ºC for 20s, 54.1ºC for 30s, 72ºC for 1min; 35 cycles of: 94ºC for 20s, 60.1ºC for 30s, 72ºC for 1min; 72ºC for 7min	302	758 – 3'(CGG)n	Fw: 5'- AGGAAGAGAGTAA GAG GGT TTT AGG TTT TTT TTG G-3' Rv: 5'- CAGTAATACGACTCACTATAGGGAGAAGGCT AAA ACA TAT ACA TTC CTA AAT TTA CCC C-3'
**FREE2 (D)**	15 mins at 95°C; 10 cycles of: 94ºC for 30s, 56ºC for 30s, 72ºC for 1min; 35 cycles of: 94ºC for 30s, 62ºC for 30s, 72ºC for 1min; 72ºC for 7min	374	902– 3'(CGG)n	Fw: 5'- AGGAAGAGAGAAAAGTTTTAGGAAGATTTTAATATGG-3' Rv: 5'- CAGTAATACGACTCACTATAGGGAGAAGGCT AAAAAACACAATAAACCCA TAAATACC-3'
**FREE2 (E)**	15 mins at 95°C;	360	1418– 3'(CGG)n	Fw: 5'- AGGAAGAGAGGAATGGTTTGAATGTTTTAGATAGGAT - 3'

	10 cycles of: 94ºC for 30s, 56ºC for 30s, 72ºC for 1min;<br><br>35 cycles of: 94ºC for 30s, 62ºC for 30s, 72ºC for 1min;<br><br>72ºC for 7min			Rv: 5'- CAGTAATACGACTCACTATAGGGAGAAGGCT ACCAAAAATCTAATAACCAAAACCAC-3'
**FREE3**	15 mins at 95°C;<br><br>10 cycles of: 94ºC for 30s, 56ºC for 30s, 72ºC for 1min;<br><br>35 cycles of: 94ºC for 30s, 62ºC for 30s, 72ºC for 1min;<br><br>72ºC for 7min	327	9908–<br><br>3'(CGG)n	Fw: 5'- AGGAAGAGAGTTTTTTTTATATAGGTATTTGTAAAGGATG -3'<br><br>Rv: 5'- CAGTAATACGACTCACTATAGGGAGAAGGCT TCTCTAATTTCTTTCTTCACATTCAAAA -3'

Note: The in vitro transcription related sequences for EpiTYPER based methylation analysis are in blue with forward primer sequence being AGGAAGAGAG and reverse primer sequence being CAGTAATACGACTCACTATAGGGAGAAGGCT

Table S6. Details of the post-mortem brain tissue processing, age and the clinical phenotype.

	Post-mortem interval (hrs)	Age of death	Clinical	Frontal cortex (mg)	Cerebellum (mg)
**FM male CASE 1**	40-44	74	FSIQ 40 at 65 years	500	
**FM male CASE 2**	20	57	Autism Disorder	500	
**FM male CASE 3**	11.5	64	FSIQ 57 in adulthood	500	
**PM/FM male CASE 4**	16	25	FSIQ 51 at 24 years; no autism	500	
**Control male CASE 1**	22	73.5	Control	91	
**Control male CASE 2**	33	52.1	Control	101	
**Control male CASE 3**	24	64.1	Control	81	
**Control male CASE 4**	42.5	72.6	Control	113	
**Control male CASE 5**	39	56	Control	106	
**Control male CASE 6**	31.5	63.9	Control	125	
**Control female CASE 1**	30	59	Control	92	123
**Control female CASE 2**	26.5	73.7	Control	111	125
**Control female CASE 3**	25	71.3	Control	119	117
**Control female CASE 4**	30.5	63.4	Control	113	139
**Control female CASE 5**	19	78.8	Control	92	85
**Control female CASE 6**	24	67.3	Control	105	110
